# Improvement of treatment adherence with growth hormone by easypod™ device: experience of an Italian centre

**DOI:** 10.1186/s13052-018-0548-z

**Published:** 2018-09-27

**Authors:** Maria Cristina Maggio, Beatrice Vergara, Paolo Porcelli, Giovanni Corsello

**Affiliations:** 1 University Department Pro.Sa.M.I. “G. D’Alessandro”, University of Palermo, Palermo, Italy; 2 Unit of Endocrinology, “Ospedali Riuniti Villa Sofia-Cervello”, Palermo, Italy

**Keywords:** R-hGH, Easypod™, Growth hormone deficiency, Treatment adherence, Small for gestational age, Turner syndrome

## Abstract

**Background:**

One of the most important vulnerabilities falling the efficacy of recombinant human growth hormone (r-hGH) treatment is low adherence especially in young patients. This study was planned to describe the correlation between r-hGH treatment efficacy and adherence in real-life setting using easypod™.

**Methods:**

Forty patients younger than 18 years, affected by a clinical condition in which r-hGH is available and treated with r-hGH easypod™, were enrolled in a retrospective, observational, real-world data, monocentric trial. The study design provided the retrospective collection of records collected by a questionnaire proposed to the patients and their parents and compared with registered data by the new generation electronic device r-hGH easypod™. Number of injections and doses were collected and used to assess the percentage of administered GH doses to measure treatment adherence. The r-hGH treatment efficacy was evaluated comparing standard deviation score for height (SDS) between baseline and follow-up visit, according to clinical practice.

**Results:**

The mean treatment adherence was 92.20% and it was inversely related to patients’ age (*R* = − 0.358, *p* = 0.023), and significantly higher in the sub-group of patients with age between 10 and 13 years. Treatment adherence showed an inverse correlation with the years of therapy (*R* = − 0.453, *p* = 0.003) and with the number of r-hGH administrations (*R* = − 0.392, *p* = 0.012). However, the height increase did not reach a significant correlation with treatment adherence (*R* = − 0.067, *p* = 0.683).

**Conclusions:**

Children and adolescent patients with GH deficiency due to different clinical conditions show high adherence to r-hGH treatment tested by easypod™. Easypod™ could be used as an important device to control patients’ adherence in daily treatment for chronic diseases with expensive drugs.

## Background

Recombinant human growth hormone (r-hGH) is widely employed for treatment of several growth disorders since 1985 [1, 2]. In Italy, r-hGH is indicated in GH deficiency (GHD), Turner syndrome (TS), short stature associated with SHOX aploinsufficiency, short children born small for gestational age (SGA), chronic renal failure (CRF) and Prader-Willi syndrome [3]. This treatment is primarily used in childhood and adolescence and the growth improvement measurement is generally used to evaluate therapy efficacy. Although it is well known that an early r-hGH treatment start improves the final height, the lack of treatment adherence could hamper the growth potential [4, 5]. Thus, treatment adherence remains the most important factor influencing the successful outcomes, as well as in the vast majority of chronic therapies [6]. Indeed, low adherence is a significant factor determining reduced growth gain with r-hGH treatment and increased health costs [7].

Chronic long-term treatments are generally characterized by low adherence. Once more, r-hGH treatment is often burdened by suboptimal adherence, especially in paediatric population. Several studies reported that up to half patients are generally not fully adherent [8]. This therapy requires daily subcutaneous injections and children often have a significant load related to the daily administration of r-hGH, missing the final goal of benefits in height [4, 5]. This discrepancy, generally called “treatment fatigue”, could negatively influence treatment adherence especially in children and adolescents [9]. Several strategies have been proposed so far in the literature to improve patients’ adherence and enhance self-administration [8]. The improvement device simplicity, convenience, appropriate education and training of both patients and parents are mandatory to increase the adherence [10]. However, the main clinical challenge remains the impossibility to accurately measure the treatment adherence [9, 11]. The accurate monitoring of the therapy remains the main objective of new therapeutic devices [6]. Indeed, a complete evaluation of patients’ adherence allows the clinicians to exclude poor adherence from the possible reasons for sub-optimal growth response, driving further treatment adjustment and exact interval among follow-up visits.

Currently, several devices are commercially available and r-hGH compounds have been improved with the replacement of traditional syringes with needles with more user-friendly devices [12]. These innovations have sought to improve adherence by simplifying injection, reducing pain, improving convenience and patient acceptability [13–16]. The electronic auto-injector device, easypod™, owns several features which improve the device simplicity, comfort and convenience, such as pre-set dosing, adjustable injection settings, an electronic skin sensor able to distinguish the skin of the patient from skin of others [17]. Moreover, easypod™ allows an accurate surveillance of treatment adherence, an injection diary that automatically registers injection times [17]. These data are available by patients and by the clinicians who follow the patients using a remote access [17].

Despite these efforts to reduce treatment discomforts and challenges, the adherence to r-hGH treatment remains suboptimal in paediatric population, due to lack of a reliable method able to comprehensively evaluate all possible causes of poor adherence. The primary objective of this study was the evaluation of r-hGH treatment adherence, using easypod™ in children and adolescents during follow-up. Secondary endpoints were: the study of r-hGH efficacy in growth improvement and height gain, considering both efficacy and treatment adherence; to limit waste involved in expensive therapies for the national health system; to optimize GH therapy, improving the quality of patient assistance.

## Methods

### Study design

A retrospective, observational monocentric study based on real-world data was carried out at the Paediatric Clinic of the “G. Di Cristina” Children Hospital, ARNAS, Palermo, Italy. All children and adolescents treated with r-hGH from 2009 to 2016, using the electronic device easypod™ were enrolled. Accordingly, r-hGH treatment and dosage were provided following the clinical practice. Patients with age below 18 years and a confirmed diagnosis of GH deficiency were enrolled, using two GH stimulating tests executed at least at the interval of 1 month. Moreover, patients in which the r-hGH treatment is approved were enrolled, such as isolated GH deficiency, SGA, chronic renal failure and Turner syndrome.

Both patients and their parents were informed about the study scope and design. The informed consent was provided to all patients and parents.

The study design provided the retrospective collection of data recorded in the electronic device easypod™ from 2009 to 2016. The number of injections and the doses were collected and analysed to calculate treatment adherence percentage, using the following formula:

$$ \frac{number\ of\ in jections\ in\ the\ time- frame}{number\ of\ in jections\ expected\ in\ the\ same\ time- frame}\times 100. $$

Data were collected at baseline, i.e. before the treatment start, and after appropriate follow-up, which was variable for each patient, according to clinical practice.

### Satisfaction questionnaire

A simple questionnaire of satisfaction was created ad hoc evaluating the several items related to the treatment (Table 1).

**Table 1:** Questionnaire of satisfaction created ad hoc for the trial

1	Quality and completeness information received prior to treatment
2	Degree of parental education
3	Ease of use of easypod™
4	Pain related to injection
5	Person who perform the injection
6	Number of injections not performed in the last moth
7	Reasons of low adherence
8	Whether patients refuse one or more of the sites proposed for injections
9	Whether patients had ever thought to abandon the therapy

### Anthropometrical parameters

The treatment efficacy was evaluated using the height gain at the follow-up, considering the change in the standard deviation (SD) of height from baseline.

Height was measured using Harpenden stadiometer (Crosswell, Crymych, Pembs., SA41 3UF, UK). Percentile of height was evaluated using the Italian growth charts available in the literature [18], growth velocity using Tanner’s charts [19], whereas skeletal age through the Greulich-Pyle method [20].

### Hormonal measurements

IGF-1 serum levels were measured to evaluate the treatment efficacy through a single radio-immunological assay. Thyroid stimulating hormone (TSH), free-triiodiothyronine (fT3), free-tetraiodiothyronine (fT4), insulin, glucose, blood counts and liver and renal function were measured to consider the treatment safety.

### Statistical analysis

Patients were classified by gender, age and treatment duration.

Correlation between the treatment adherence and parameters collected was performed using Pearson’s correlation. Treatment efficacy was evaluated considering the SD of height at baseline and at the end of follow-up.

Sub-groups analyses were performed dividing patients according age in three groups: 14 patients were between age 5 and 9 years, 15 patients were between age 10 and 13 years, and 11 patients were with age above 14 years. Moreover, patients were divided in group A (seven administrations weekly) and group B (six administrations weekly), according to the treatment schedule. Differences among sub-groups of age were evaluated by Fisher exact test.

All variables were tested for normal distribution using the Anderson-Darling normality test. All variables were expressed as mean ± standard deviation.

Pearson product moment correlation coefficient was employed to calculate the degree of linear correlation between the parameters analyzed (auxological, clinical, personal records reported in the questionnaire, hormonal data).

Statistical significance was considered with *p* less than 0.05. Calculations were done using “MiniTAB release 13.1 Statistical Software”.

## Results

Forty young patients treated with r-hGH were enrolled, with a mean age of 11.2 + 2.3 years (Table 2). The group of patients consisted of 27 males (67.5%) and 13 females (32.5%). The 75% of patients were prepubertal at the time of enrolment, whereas the 25% of cases had been started the puberty at baseline. Twenty-six patients showed isolated GH deficiency (65%), 9 were SGA (22.5%) and 5 girls were affected by Turner’s syndrome (12.5%). R-hGH treatment was started between 2.51 and 15.1 years of age (mean age 8.65 + 2.81 years).

**Table 2:** Comparison between the parameters at the start and at the end of treatment

	Baseline evaluation	Follow-up evaluation	*p*-value
Age of start therapy (years)	8.65 + 2.81	11.17 + 3.0	*0.023*
Years of therapy		2.41 + 1.86	–
SDS of height	−2.56 + 1.10	−1.96 + 1.24	> 0.05
Growth velocity (cm/year)	4.10 + 1.17	6.76 + 3.02	*0.044*
Target height	160.51 + 6.64		–
Bone age (years)	7.15 + 2.84	11.44 + 4.10	*0.010*
IGF-1 (ng/ml)	168.20 + 99.20	320.70 + 151.20	*0.032*
Percentage of adherence	–	92.20 + 8.90	–
Missing doses/month	–	1.20 + 1.35	–

Significant entries were italicized

### Treatment adherence

The mean treatment adherence was 92.2% and it was inversely related to patients’ age (*R* = − 0.358, *p* = 0.023), and significantly higher in sub-group of patients with age between 10 and 13 years, compared to those with age below 9 years or above 14 years (*p* < 0.001; 96.43% versus 92.42% and 82.28%, respectively).

**Fig. 1:**
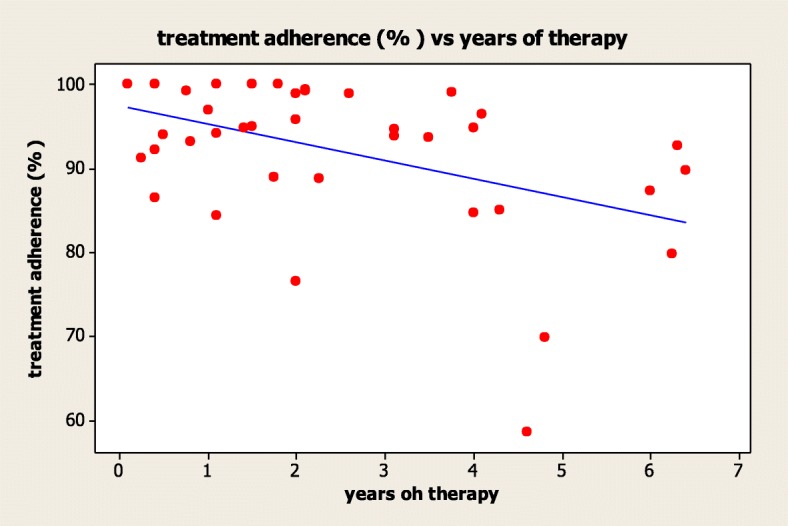
Linear correlation between adherence and years of therapy

Treatment adherence was inversely related to the years of therapy (*R* = − 0.453, *p* = 0.003) (Fig. 1).A higher adherence was found in patients treated for 1 year (*n* = 13, 96.0%) and in those treated for 2–4 years (*n* = 17, 94.7%), compared to patients treated for more than 4 years (*n* = 10, 83.9%), although not significantly different (*p* = 0.385). moreover, treatment adherence was similar between patients who started treatment before puberty (*n* = 33, 92.23%) and those who started treatment after the puberty onset (*n* = 7, 92.0%) (*p* = 0.956).

**Fig. 2:**
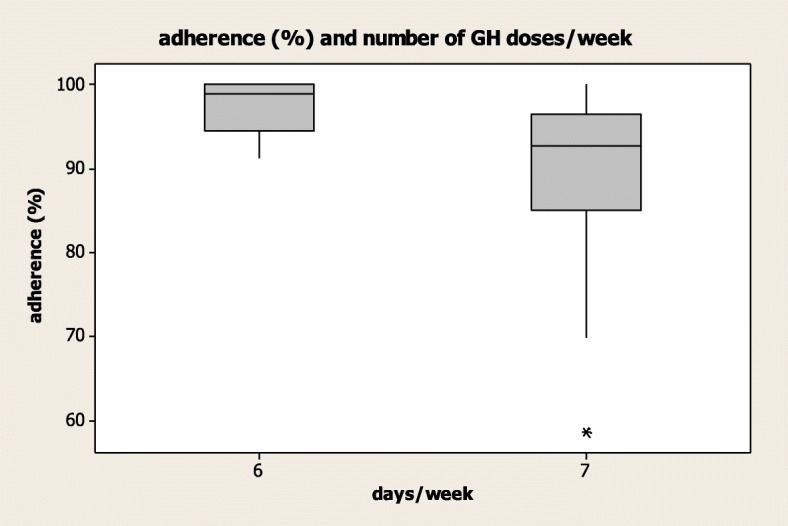
Linear correlation between adherence (%) and the number of r-high doses

Patients treated with injections for 6 days a week showed a higher treatment adherence (97.26%) compared to patients treated with injections for 7 days a week (89.8%). The mean treatment adherence was inversely related to the number of r-hGH doses (*R* = − 0.392, *p* = 0.012) (Fig. 2).

### Treatment efficacy

A final height increase after a mean follow-up of 2.41 + 1.86 years was detected, although not statistically significant (*p* > 0.05) (Table 2). Particularly, the average height increase was 0.61 SD, ranging from − 2.56 + 1.10 SD to − 1.95 + 1.24 SD (Table 2). Moreover, a significant growth velocity increase was demonstrated after follow-up (*p* = 0.004) (Table 2). However, the height gain was neither related to the patients’ age, nor to the age at treatment start (*R* = 0.301, *p* = 0.059). The improvement of height was directly related only to parental height target (*R* = 0.487, *p* = 0.002).

**Fig. 3:**
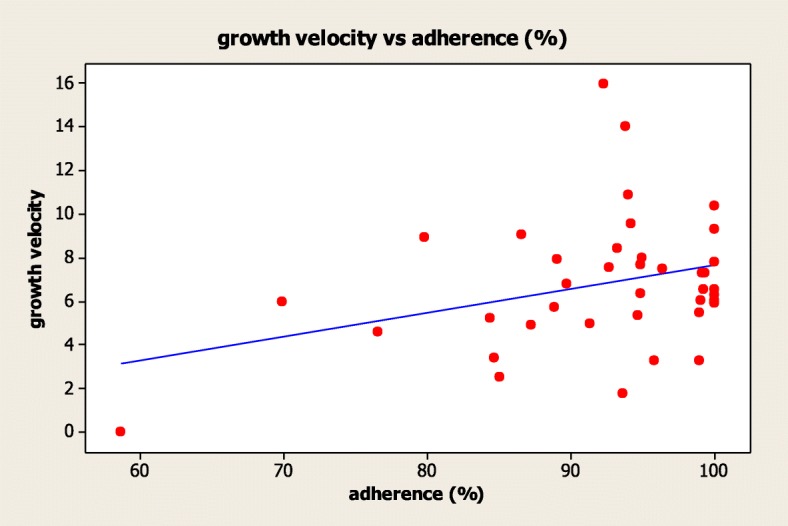
Correlation between growth velocity and treatment adherence

The treatment efficacy, considered as height gain during r-hGH treatment, did not correlate with treatment adherence (*R* = − 0.067, *p* = 0.683). Treatment adherence showed a significant correlation with IGF-1 serum levels (*R* = − 0.398, *p* = 0.032), which did not correlate with the final height and growth velocity (*R* = 0.184, *p* = 0.340 and *R* = 0.161, *p* = 0.422, respectively). Finally, growth velocity was directly related to treatment adherence (*R* = 0.325, *p* = 0.044) (Fig. 3), showing that children with a higher adherence showed a higher growth velocity.

### Questionnaire evaluation

The totality of parents and patients declared to receive clear and complete information regarding the treatment scope and modality of administration (100%). Similarly, all patients and parents stated that easypod™ was easy to use (100%).

Comparing the electronic evaluation of adherence, with the questionnaire answers, 26 patients (65.0%) referred a lower number of skip doses compared to what registered by easypod™, on the contrary 5 patients (12.5%) referred a higher number. Thus, 9 patients (22.5%) referred a skip doses number equal to what registered by the electronic device. In general, the mean skip doses number referred to parents was 1.3 doses monthly, although increasing until 2.5 doses monthly considering easypod™ data. This result suggests the importance of use an electronic device able to objective data regarding therapy compliance. Considering the reasons for skip doses, they occurred mainly because of family journey (*n* = 7), followed by tiredness (*n* = 6), forgetfulness (*n* = 5), illness (*n* = 5), exhaustion needles or devices (*n* = 4), and technical problem to easypod™ (*n* = 2). The 35% of patients treated (*n* = 14) referred bother or discomfort at site of injection, whereas only the 5% (*n* = 2) referred pain. The pain degree seemed to be inversely related to the treatment adherence, although not statistically significant (*R* = − 0.289, *p* = 0.071).

**Fig. 4:**
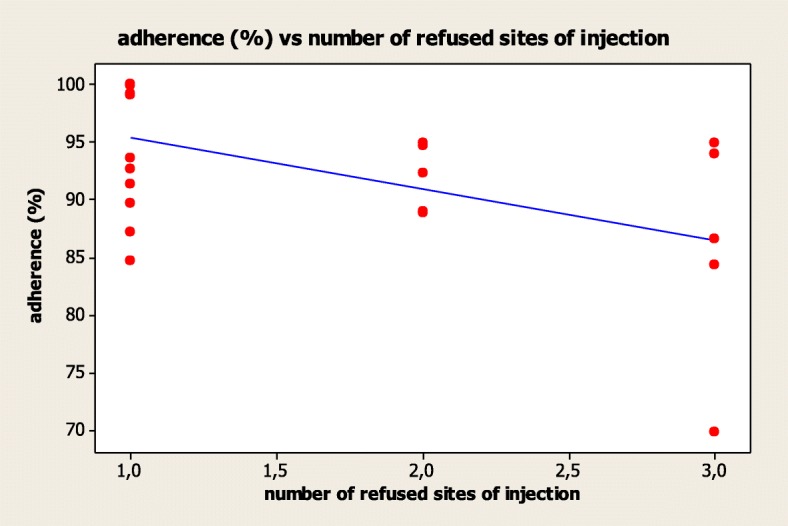
Correlation between adherence (%) and the number of refused sites of injection

The r-hGH administration was performed by patients in 17.5% (*n* = 7), by parents in 65.0% (*n* = 26) and by both patient and parents in 17.5% (*n* = 7). The adherence was significantly lower in those patients who self-injected r-hGH (80.9%), compared to those treated by parents (95.2%) (*p* = 0.002). Only the 37.5% (*n* = 15) of patients used all possible sites of injection, whereas the 32.5% (*n* = 13) refused at least one site (generally abdomen), 12.5% (*n* = 5) refused two sites and 17.5% (*n* = 7) used only one of the possible sites of injection. The number of refused sites of injection was inversely related to the treatment adherence (*R* = − 0.520, *p* = 0.011) (Fig. 4). Finally, the parents’ “level of education” did not show a statistically significant correlation with the treatment adherence.

Considering the overall satisfaction, 24 patients stated a high satisfaction for the achieved results (60.0%). On the contrary, 12 patients declared a mild satisfaction (30.0%), 3 a poor satisfaction (7.5%) and only one patient was dissatisfied by the treatment (2.5%).

## Discussion

This retrospective, observational study based on real-life data shows the experience of a single Italian Centre in the treatment of GH deficiency in young patients using easypod™ electronic device. The study demonstrated a high r-hGH treatment adherence in childhood and adolescence. The device automatic report shows an average adherence of 92.2%, which is significantly higher than the minimal percentage proposed to define the good adherence to r-hGH administration (85%) [21]. Moreover, the data registration by the electronic device allows at demonstrating that 35% of patients erroneously relays their treatment adherence. Thus, easypod™ allows to obtain a high adherence with a low percentage of relay errors. Accordingly, Bozzola et al. previously found an average adherence to r-hGH administration of 87.5% in 824 children, with a concordance between data collected by easypod™ and data relayed by patients/parents in the 84.3% of the cases [22]. Moreover, data from an American GH registry based on physician-reported adherence, indicate that the 76–85% of patients missed 0–3 doses per month [4]. All these results confirm the importance to measure adherence by the electronic device, bypassing potential relayed errors. Easypod™ represents one of the most recent advances obtained in the field of r-hGH therapy, which could be optimal to obtain these achievements. Moreover, all patients and parents enrolled in this study consider the device to be quick, easy and comfortable to use.

All the patients and the parents were encouraged to r-hGH treatment by physicians, explaining them the benefits of r-hGH treatment on growth and global health. They were encouraged to discuss with the physicians about their fears and troubles, linked to r-hGH treatment, at every control visit. The impact of socioeconomic level and the parents’ instruction degree, evaluated by a point ad hoc included in the questionnaire, were minimized by the simple language of the questionnaire and by the personalized trial; the latter has been dedicated to each naïve patient and to their parents and it was offered by a professional and specialized nurse.

Despite these improvements, the treatment adherence is not complete, leaving challenges at identifying the multifaceted reason for poor adherence [23].

This study confirms the difficulties to treat chronic diseases in childhood and adolescence. However, an inverse relationship between adherence and patient’s age is debated. Increasing the patient’s age, a decrease adherence is found, suggesting the difficulties to manage GH deficiency especially during adolescence. This confirms the difficulty of adolescents to accept the idea to suffer a chronic disease, which makes them “different” from peers. In particular, during adolescence, patients start to take care of their own health, they must accept their underlying disease and they come from a long-time therapy during childhood [5]. All these challenges should be considered in the management of r-hGH treatment. In our group, the most adherent patients belonged to the age 10–13 years, whereas a reduced adherence found in both older and younger patients [5]. However, the percentage of adherence progressively decreases, increasing the treatment period. The treatment with the same dose, divided into 6 days every week, seems to be better tolerated than a complete weekly protocol. Thus, a comprehensive evaluation of patients’ setting and therapeutic schemes available should be mandatory to improve both adherence and efficacy in clinical practice.

Several prospective interventional studies previously demonstrated the r-hGH efficacy in idealized and rigorously controlled conditions [1, 2]. However, it is well known that interventional studies show high internal validity, even if external one is reduced and data should be confirmed in the clinical practice [24]. We performed an observational study based on real-life approach to GH deficiency in childhood and adolescence, using real-world data, confirming the r-hGH efficacy and effectiveness, with a mean height increase of 0.60 SD compared to the baseline height. Although this increase is not statistically significant, it is in line with the definition of a good r-hGH treatment response, which is reached when the height increase is more than 0.3–0.5 SD [25, 26]. Thus, we confirm the efficacy of r-hGH administration in our patients with isolated GH deficiency, SGA, chronic renal failure and Turner syndrome, even if we cannot divide our patients in different groups, according to the primary disease, for the numerically reduced numbers of some subgroups. However, in our patients the height gain is not related to treatment adherence, differently to previous studies using easypod™ [27], although it correlates with IGF-1 serum levels.

Patients started puberty at different timing of the treatment period; some patients started at the beginning of therapy, some patients after some years and could have influenced the statistical significance of the height gain during the therapy. These differences could have influenced growth in GH deficient patients, whose diagnosis was done in line with international protocols. All the patients showed a pathological short stature, low growth velocity, delayed bone age and documented by two pathological tests (GH < 8 ng/ml).

These results could be explained by the high rates of adherence, above 85%, found in the study, which guarantees an appropriate growth velocity. Accordingly, IGF-1 significantly increased during treatment, remaining within the normal range. The final height shows a significant correlation with target height, suggesting a possible main role of parental height in the setting of GH deficiency, although it is controversial in the literature. Indeed, some studies demonstrated a higher final height after r-hGH treatment compared to the target in congenital adrenal hyperplasia [28]. On the contrary, no correlations were found in Prader-Willi syndrome [29]. Further studies properly designed are needed to better understand the role of the genetic background on final height, as shown in children with GH deficiency associated with SHOX deficiency [30].

Finally, the highest treatment adherence was reached when the dose was provided by parents, when no pain occurred and when all sites of injection were selected. These results derived from questionnaire ad hoc designed to screen r-hGH treatment satisfaction, suggesting that parents and patients need information and training to improve the treatment adherence. Once again, the multifactorial characteristics of treatment adherence are confirmed, in the chronic daily r-hGH therapy. However, a pharmaco-economy evaluation should be necessary in this setting, to compare the r-hGH doses used, the missed doses and the efficacy of the treatment. To this purpose, an electronic device able to recognise patients with poor adherence is fundamental in the global strategy aiming at bypassing all challenges in r-hGH therapy.

## Conclusions

The strength of our study is characterized by the evaluation of both efficacy and adherence of r-hGH treatment using a real-life monocentric approach. Moreover, the complete evaluation of adherence using both questionnaires and electronic devices, allows at confirming the multifactorial nature of treatment adherence.

The injection-recording system and the possibility to monitor the real recipient of the treatment by easypod™ could enhance the ability of the physicians to monitor adherence to r-hGH.

Furthermore, r-hGH treatment is an expensive therapy, guaranteed in Italy by the national health system for the treatment of the authorized diseases. This condition requires an accurate surveillance with the finality of reducing waste and of containing costs. These goals significantly contribute to optimize r-hGH treatment and to allow this expensive therapy to all the patients who need it.

The questionnaire proposed to our patients could be used to increase surveillance on r-hGH treatment adherence and to prevent potential causes of reduced compliance. However, the discordance between the questionnaire answers and the easypod™ records induce to choose the electronic device to obtain a real report of administered doses.

However, the main limits of our study are the low number of enrolled patients and the lack of a control group. For the limited number of patients with diseases other than GH deficiency, the comparison of compliance with r-hGH treatment in subjects affected by different diseases could not be statistically evaluated.

Moreover, both patients and parents are aware that adherence is monitored during the study, and that a good adherence to the therapy influences the final height. Future larger multicentre observational trials based on real world data should be useful to better understand and describe the effectiveness and the adherence of r-hGH treatment.

In conclusion, easypod™ is an electronic device able to monitor the adherence to daily r-hGH treatment in children and adolescent with GH deficiency as well as in patients who need other expensive treatment self-managed by the family at home, which need adherence and surveillance.

## Abbreviations

CRF: Chronic renal failure
GHD: Growth Hormone deficiency
GHD: r-hGH: recombinant human growth hormone
SDS: Standard deviation score
SGA: Small for gestational age.
TS: Turner Syndrome
